# The Incidence and Variants of the Reno-Hemiazygos Connection

**DOI:** 10.3390/diagnostics15040441

**Published:** 2025-02-11

**Authors:** Nawwaf Sebastian Damen, Adelina Maria Jianu, Mihai Lazăr, Mugurel Constantin Rusu, Gabriel Piţigoi, Silviu Petrescu

**Affiliations:** 1 Department of Anatomy and Embryology, Faculty of Medicine, “Victor Babeș” University of Medicine and Pharmacy, 300041 Timișoara, Romania; nawwaf.damen@umft.ro (N.S.D.); adelina.jianu@umft.ro (A.M.J.); 2 Department 2, Division of Physiopathology II, Faculty of Medicine, “Carol Davila” University of Medicine and Pharmacy, 020021 Bucharest, Romania; mihai.lazar@umfcd.ro; 3 Division of Anatomy, Department 1, Faculty of Dentistry, “Carol Davila” University of Medicine and Pharmacy, 020021 Bucharest, Romania; 4 Department 9—Physical and Rehabilitation Medicine, Faculty of Medicine, “Carol Davila” University of Medicine and Pharmacy, 020021 Bucharest, Romania; gabriel.pitigoi@umfcd.ro (G.P.); silviu.petrescu@umfcd.ro (S.P.)

**Keywords:** renal artery, renal vein, hemiazygos vein, ascending lumbar vein, kidney

## Abstract

**Background/Objectives**: Although common anomalies of the left renal vein (LRV) are pretty well documented in the literature, the drainage of the left renal blood via the hemiazygos vein lacks comprehensive support. We, therefore, aimed to study the incidence of the reno-hemiazygos connection (RHC). **Methods**: A total of 150 computed tomography scans (85 men and 65 women) were documented for the origin of an RHC from the LRV. **Results**: RHCs were found in 14/150 cases (9.34%). They were more prevalent in women (71.43%). In 11/14 cases, type 1 RHCs ascended directly along the postero-lateral left side of the aorta (direct hemiazygos flow). In 3/14 cases, type 2 RHCs (indirect hemiazygos flow) had a lumbar segment corresponding to the second lumbar vein and a pretransversary segment corresponding to an ascending lumbar vein. In 9/14 cases (64.29%), the RHC was connected to a typical LRV. In 1/14 cases (7.14%), the RHC was connected to the junction between the LRV and a left retropelvic tributary. In another case (7.14%), the RHC was connected to a retroaortic LRV and, in three cases (21.43%), to a circumaortic LRV. Triple left renal arteries were found in type 1 and, respectively, type 2 cases. The vertebral level of the inferior end of the RHC was variable, from the L1/L2 disc level to the L3 level. **Conclusions**: When present, the RHC serves to connect the superior and inferior caval systems. This may be physiologically of use or not, but surgically, it is a major anatomical risk factor for bleeding if its presence is not checked preoperatively.

## 1. Introduction

Surgeons routinely perform operative procedures in the retroperitoneum without complications, but variations in the specific venous anatomy could lead to life-threatening situations [1]. Information about the renal vascular pedicle’s location and composition is essential for vascular surgeons, general surgeons, traumatologists, urologists, and radiologists [2].

The left renal vein (LRV) is commonly longer than the right [3]. The LRV passes in front of the aorta, just below the origin of the superior mesenteric artery and above the horizontal duodenal segment, ending in the inferior cava vein [3]. The LRV receives the left gonadal and ureteral veins and, generally, the left inferior phrenic and the left suprarenal veins [3,4]. The typical anatomical variations of the LRV are the retroaortic LRV (RLRV) and the circumaortic LRV (CLRV) [5,6,7]. The variable number of renal veins (RVs) and renal arteries (RAs) is also a common finding [6,8]. Commonly, RV anomalies are clinically silent [9]. Anatomic variants of the LRV can be diagnosed by ultrasound, computed tomography (CT), or magnetic resonance imaging (MRI) [10]. Experience has shown us that the renal vessels’ anatomical variations can be accurately studied three-dimensionally using angioCT files [5].

Typically, the hemiazygos vein (HAzV) continues the left ascending lumbar vein (ALV) and further receives the subcostal vein and the posterior intercostal veins 9–11; it finally drains into the azygos vein, usually at the level of T9–T10 [11]. Communicating vessels connecting the adrenal and azygos system of veins are found on the left side only [12].

In an anatomical report, Pallangyo et al. (2016) observed that although common anomalies of RVs are pretty well documented in the literature, the drainage of renal blood via the azygos venous system lacks published support [13]. A meta-analysis of the prevalence of RV variants classified CLRVs according to Gillot’s (1978) description in which, in type 1, the retroaortic arm of the CLRV is joined by the root of the HAzV [6,14].

Rodin et al. (1996) determined the incidence and morphologies of anomalous LRVs by MR resonance venography because the available literature regarding MRI appearances of LRV anomalies was limited to case reports [15]. They found CLRVs and an RLRV, but the anastomosis of the HAzV with the LRV was not found or reported. Conventional ultrasonography of the kidney is not generally helpful in evaluating renal vessels [16]. Such information can be obtained using Doppler techniques [16].

We recently found a male case with an RHC connection to an RLRV [5]. The RLRV had two caval ends [5]. The RHC had a medial root inserted into the upper caval end of the RLRV and a lateral root inserted into the RLRV main trunk [5]. Therefore, we aimed to study the incidence of this overlooked anatomical variation by including these previous findings in the present study. CT angiography is a highly accurate method for evaluating anatomical variations of the renal vessels [17].

## 2. Materials and Methods

A retrospective sample of archived DICOM angioCT files of 150 adult cases, 85 men and 65 women, was used to study renal vessels. Inclusion criteria were good quality scans, complete filling of the renal vessels (arteries and veins), full scans of the lower thorax, abdomen and pelvis, and no pathological processes to distort or blur the renal vascular anatomy. Exclusion criteria were inadequate scans to observe the anatomy of the abdominal vessels, pathologic processes distorting the anatomical features, and previous retroperitoneal surgery. No cases were excluded. Our study was aligned with the principles of the Declaration of Helsinki (World Medical Association Code of Ethics). The Ethical Committee of the “Victor Babeş” University of Medicine and Pharmacy of Timişoara, Romania (affiliation 1) approved this study (approval no. 16178/11 July 2023).

As in previous studies, the CT angiograms were performed with a 32-slice scanner (Siemens Multislice Perspective Scanner) and the renal vascular variants were documented using the Horos 3.3.6 (Horos Project, Annapolis, MD, USA) program [5,18,19,20]. All the authors independently performed evaluations of the presence and types of RHCs. Two types of RHC were determined: type 1, direct hemiazygos flow from the LRV, and type 2, indirect hemiazygos flow from the LRV via the ALV. Each author recorded the vascular morphologies identically. An experienced anatomist (M.C.R.) and an experienced radiologist (M.L.) validated the results. The accuracy of the anatomical identification was validated by two experienced anatomists (M.C.R. and A.M.J.).

The cases with RHCs were recorded and documented individually. Data were further processed using Microsoft Excel and descriptive statistics.

## 3. Results

Out of 150 cases, 14 (9.34%) were found to have RHCs. Four RHCs were found in males (28.57%) and ten RHCs were found in females (71.43%). In 9/14 cases (64.29%), the RHC was connected to a typical LRV. In 1/14 cases (7.14%), the RHC was connected to the junction between the LRV and a retropelvic tributary (RP). In another case (7.14%), the RHC was connected to an RLRV (see Introduction) and, in three other cases (21.43%), to a CLRV. In 2/4 male cases, the RHC was connected to the LRV (50%), in another case (25.00%) to an RLRV, and in the last case (25%), to a CLRV. Out of the 150 cases, there were also 3/150 RLRV cases without RHCs and 5/150 CLRV cases without RHCs. In females (*n* = 10), the RHC was connected to the LRV in 7/10 cases (70%). Peculiarly, in 1/10 cases (10%), it connected to the junction of the LRV and the RP on that side. In 2/10 cases (20%), it connected to the posterior arm of a CLRV.

**Figure 1:**
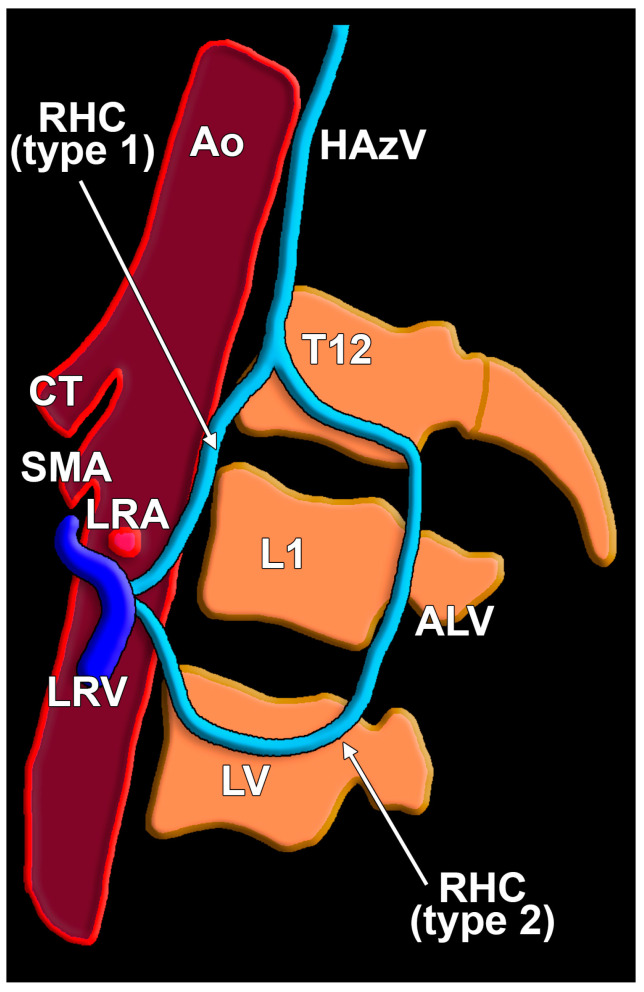
Left lateral view of a diagram depicting the two types, 1 and 2, of the reno-hemiazygos connection. ALV, ascending lumbar vein; Ao, aorta; HAzV, hemiazygos vein; LRA, left renal artery; LRV, left renal vein; LV, second left lumbar vein; and SMA, superior mesenteric artery.

**Figure 2:**
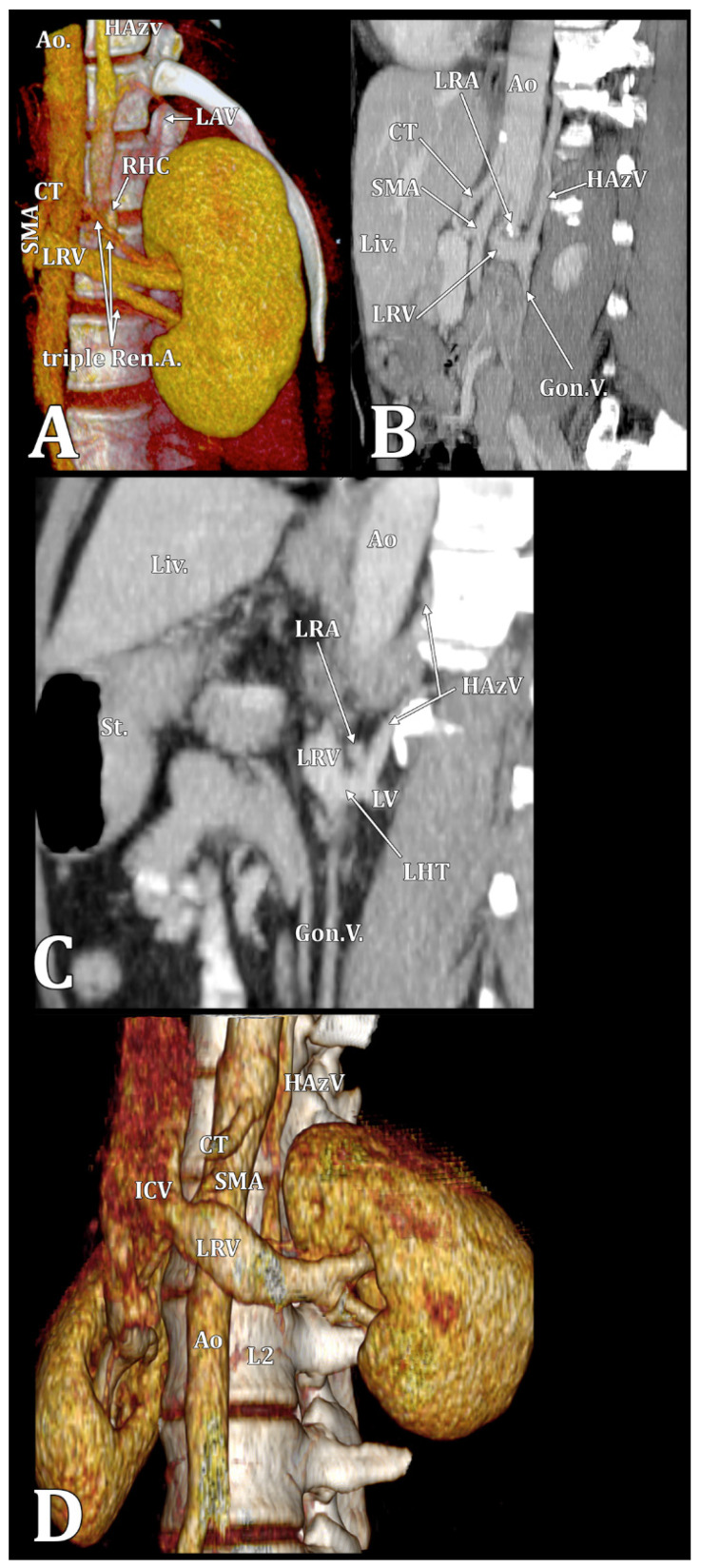
Type 1 RHC cases. (**A**) A triple left renal artery was found in a type 1 male case. In that case, the RHC had a 9.56 mm caliber, and the LRV was 9.61 mm. Therefore, the LRV and RHC were almost equal in size. The upper and middle left renal arteries crossed the RHC anteriorly. Three-dimensional volume rendering of the left kidney and renal vessels, anterior view. Ao., aorta; CT, celiac trunk; HAzV, hemiazygos vein; ALV, lumbar ascending vein; LRV, left renal vein; Ren.A., renal artery; RHC, reno-hemiazygos connection; and SMA, superior mesenteric artery. (**B**) In a type 1 female case, the LRV crossed the aorta anteriorly at the level of the L2 vertebra. Immediately after crossing the aorta, the LRV connected an RHC posteriorly and, inferiorly, the left gonadal vein. The RHC ascended posterior to the left RA and continued as an HAzV on the posterior side of the aorta. Sagittal angioCT slice. Left lateral view. Ao, aorta; CT, celiac trunk; Gon.V., left gonadal vein; HAzV, hemiazygos vein; Liv., liver; LRA, left renal artery; LRV, left renal vein; and SMA, superior mesenteric artery. (**C**) In a type 1 RHC female case, the second left lumbar vein and the RHC were inserted into the LRV as a common lumbar-hemiazygos trunk, immediately inferior to the left renal artery at the L1/L2 intervertebral disc level. Sagittal angioCT slice. Left lateral view. Ao, aorta; Gon.V., left gonadal vein; HAzv, hemiazygos vein; LHT, lumbar-hemiazygos trunk; Liv., liver; LRA, left renal artery; LRV, left renal vein; LV, lumbar vein; and St., stomach. (**D**) In a type 1 RHC female case, the HAzV had a 6.8 mm caliber. Three-dimensional volume rendering. Left antero-infero-lateral view. Ao, aorta; CT, celiac trunk; HAzV, hemiazygos vein; ICV, inferior cava vein; LRV, left renal vein; and SMA, superior mesenteric artery.

**Figure 3:**
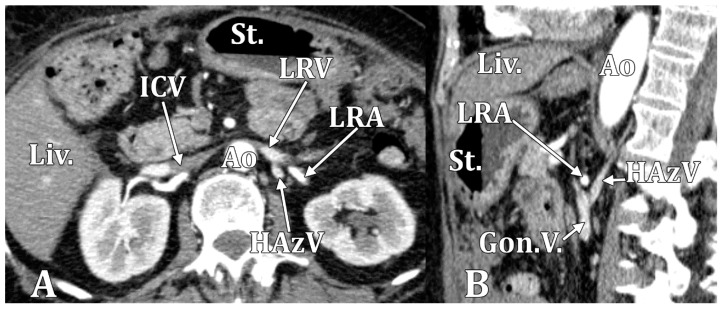
In a type 1 RHC female case, the LRV was squeezed on the anterior side of the aorta. The left gonadal vein and the HAzV appeared dilated, suggesting an anterior nutcracker phenomenon. (**A**) Axial slice, inferiorly viewed. (**B**) Sagittal slice, left lateral view. Ao, aorta; Gon.V., left gonadal vein; HAzV, hemiazygos vein; ICV, inferior cava vein; Liv., liver; LRA, left renal artery; and St., stomach.

**Figure 4:**
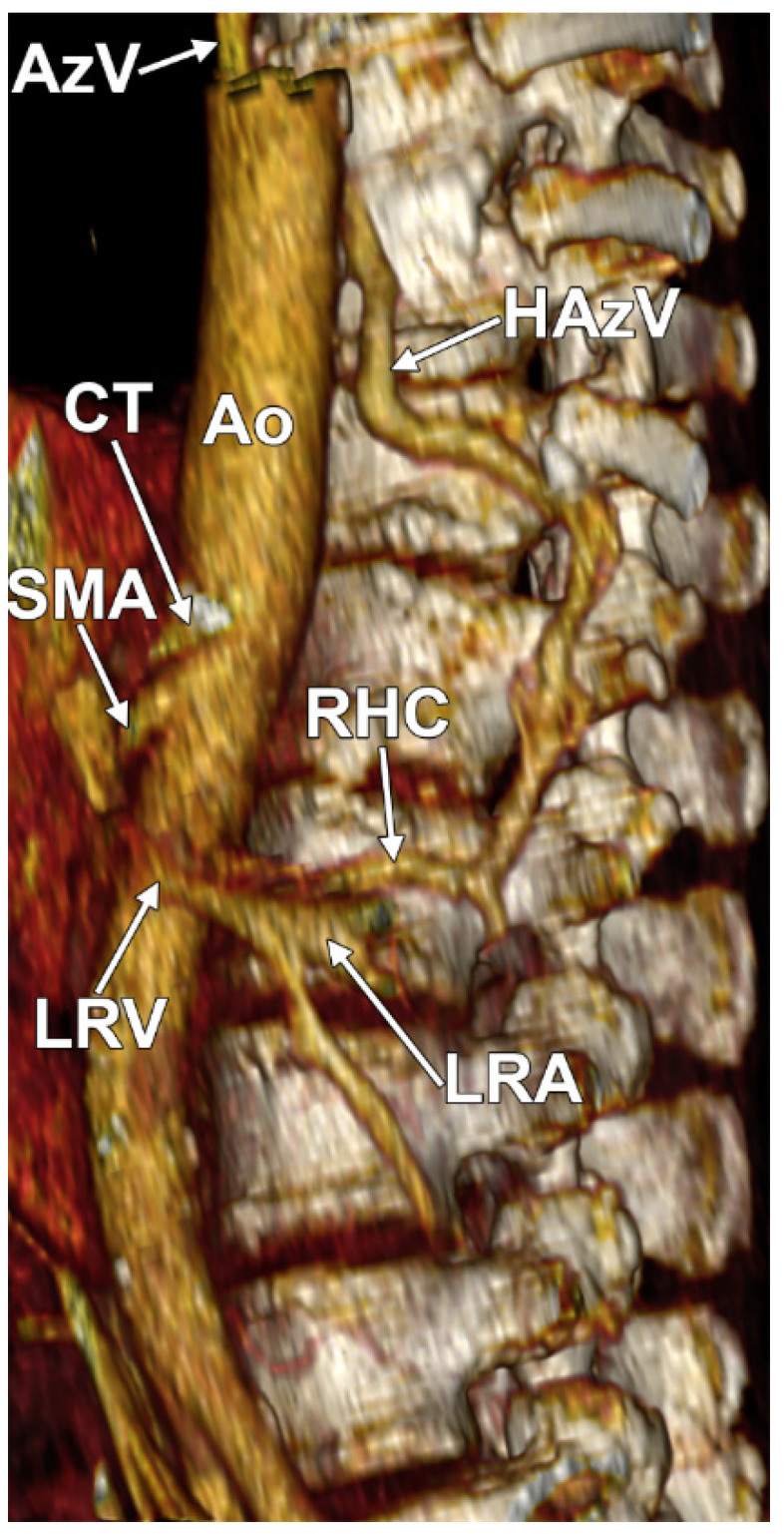
In a female case, the type 2 RHC coursed posteriorly on the left side of the L2 vertebra as a lumbar vein and ascended in front of the vertebral transverse processes of the T12-L2 vertebrae as an ascending lumbar vein. It crossed the left side of the T12 vertebral body and continued superiorly as an HAzV. Three-dimensional volume rendering. Left lateral view. Ao, aorta; AzV, azygos vein; CT, celiac trunk; HAzV, hemiazygos vein; LRA, left renal artery; LRV, left renal vein; and SMA, superior mesenteric artery.

In 11/14 cases (78.57%), the type 1 RHC ascended directly along the postero-lateral left side of the aorta (direct hemiazygos flow) (Figure 1, Figure 2 and Figure 3) and in 3/14 cases (21.43%), the type 2 RHC had a lumbar segment corresponding to the second lumbar vein and a pretransversary segment corresponding to an ALV (Figure 1 and Figure 4). The distribution of types 1 and 2 by gender is presented in Table 1. The RHC types were inserted inferiorly in different types of LRVs (Table 2, Supplemental Video S1). Triple left RAs were found in type 1 and type 2 cases. The vertebral level of the inferior end of the RHC was variable (Table 3).

## 4. Discussion

Pick and Anson (1940) clearly described that LRVs are closely associated not only with the venous drainage of neighboring organs, but also may communicate with the left lumbar veins and the HAzV (the second lumbar vein serving as an intermediary) [21]. Therefore, these connections link the LRV with the thoracic and abdominal wall veins, as well as spinal, meningeal, pulmonary, and pleural veins [21]. The two authors also observed in 68.84% of cases, an RLRV is connected with the lumbar veins, with extensive prevertebral plexuses, with the HAzV and, occasionally, the iliac veins [21].

Later, Anson et al. (1948) observed deficiencies and inaccuracies in standard works and journals relating to the RVs, and referred to a dissection study of 80 cadavers in 1888 where Lejars found a connection between LRVs and HAzVs in 88% of cases, which he termed the “reno-lumbo-azygos canal” [22,23]. The Lejars’ canal appeared as a posterior branch of the LRV, which split into “une branche lombaire et une branche destinee a l’azygos, les deux reunies en un canal commun” (transl. „a lumbar branch and a branch for the azygos, both joined into a common canal”) as quoted by Monkhouse and Khalique (1986) [12,23].

Făgărăşanu (1938), also quoted in Anson et al. (1948), defined nine types of collaterals of the LRV: (1) reno-hemiazygos; (2) reno-lumbar; (3) double reno-azygos tributaries; (4) reno-caval vein of precaval or postcaval types; (5) double canal of reno-azygos and reno-hemiazygos veins; (6) reno-azygo-lumbar canal (Lejars’ canal); (7) reno-azygo-caval canal; (8) triple canal of reno-hemiazygo-lumbo-caval veins, with iliac communications; and (9) common canal with multiple reno-genito-lumbar tributaries, with reno-genito-azygos communications [22,24]. As quoted by Pilcher and Padhani (1997), Făgărăşanu (1938) reported a 91.5% incidence of these collaterals [24,25].

The HAzV continues the ALV that courses posterior to the left renal pedicle and is the vein that could communicate with the LRV; such LRV-to-ALV anastomoses were proven by computed tomography [25]. Pilcher and Padhani (1997) documented that Davis et al. (1958) found in a dissection study that in 30% of cases, the second lumbar vein connects the ALV and LRV [25,26]. In the present study, only 3/14 cases with type 2 RHCs had a pretransversary segment of this connection superposed on the ALV’s course. In 9/14 cases, we found a direct insertion of the HAzV into the LRV. Lien and Kolbenstvedt (1977) demonstrated LRV-to-ALV communication in 34% of cases in a phlebographic study [27], but a specific HAzV filling from the LRV was neither found nor discussed. A retroaortic right RV draining into a left-sided IVC can also contribute to hemiazygos continuation, highlighting the complexity of venous drainage patterns [28].

**Figure 5:**
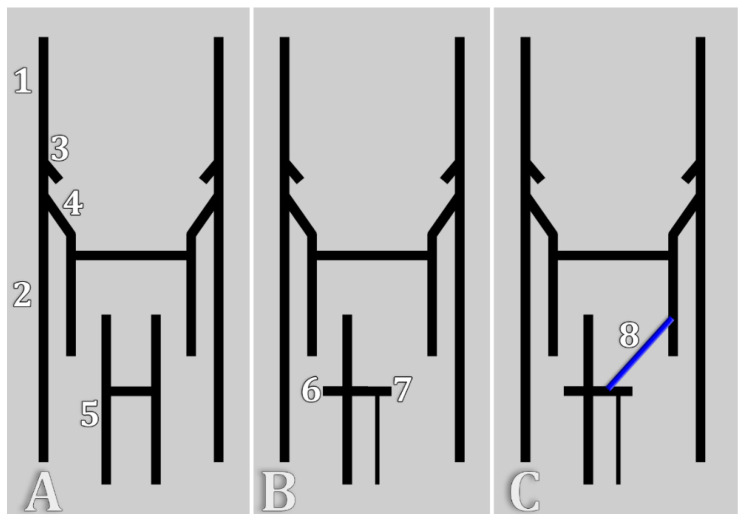
Stages of the evolution of the cardinal veins and the current report-derived hypothesis. (**A**) Bilateral symmetry of cardinal veins. (**B**) Bilateral asymmetry of the subcardinal veins. (**C**) Persistence of the proximal segment of the left subcardinal vein could explain the origin of the hemiazygos vein in the left renal vein. 1. Right anterior cardinal vein; 2. right posterior cardinal vein; 3. right common cardinal vein; 4. right supracardinal vein; 5. right subcardinal vein; 6. right renal vein; 7. left renal vein, disappeared proximal segment of the left subcardinal vein; and 8. persisting proximal segment of the left subcardinal vein—renal vein origin of the hemiazygos vein.

The body’s venous system develops from the embryonic cardinal vein system. The cardinal vein system initially consists of anterior and posterior cardinal veins. On either side of the median plane, the anterior and posterior cardinal veins form the common cardinal vein. Caudal to the venous sinus and internal to the posterior cardinal veins, supracardinal, subcardinal, and sacrocardinal veins appear later in embryogenesis. The supracardinal veins will form the azygos vein on the right and the hemiazygos vein on the left. The right subcardinal vein forms the renal portion of the IVC and the RRV. The anastomosis of the subcardinal veins forms the LRV, and then the proximal segment of the left subcardinal vein disappears, and its distal segment becomes the left gonadal vein [29]. Therefore, the insertion of the HAzV into the LRV we report here suggests the persistence of the proximal segment of the left subcardinal vein (persisting sub-supracardinal anastomosis), which allows the venous drainage of the left kidney to both the inferior and superior vena cava systems (Figure 5). When ICV and RV anomalies were reviewed on an embryological basis, the RHC was overlooked [1,30]. Bergman’s *Encyclopaedia of Human Anatomic Variation* suggests the possibility of persistence of this segment of the left subcardinal vein [31]. According to the authors, the HAzV communicates with the LRV in most individuals [31]. Still, the source of this specific information is not quoted [31]. Here, we found the RHC in just 10% of the cases.

Macchi et al. (2003) reasonably speculated that an RLRV would form if the dorsal part of the sub-supracardinal anastomosis and the intersupracardinal anastomosis persist, whereas the ventral part of the sub-supracardinal anastomosis and the intersubcardinal anastomosis regress [32].

We included the only case we found with an RLRV inserting an RHC into this lot. We also found RHCs inserted into the retroaortic arm of CLRVs. This supports both theories of persisting sub-supracardinal anastomosis, ours and Macchi et al.’s (2003) [32]. However, this theory explains cases with RLRV or CLRV but without RHCs only if a different insertion of the anastomosis into the left supracardinal vein is considered.

Different meta-analyses of the renal vessels did not document or refer to the RHC [6,8]. Neither original studies of renal vessels documented RHCs [33,34,35,36,37,38,39]. A study pf 500 patients by Arslan and Sarikaya (2022) aimed to determine the prevalence of the ALV [40]. Different variables were determined [40]. However, the HAzV was not explicitly studied [40]. The authors regarded the RHC as an LRV-connected ALV [40]. Post hoc analysis showed that an ALV was more common in patients with RLRV and CLRV than in patients with preaortic LRV [40]. In most cases, the RHCs we found in the present study were related to a preaortic LRV. In three of our cases, the type 2 RHCs had pretransversary segments that could fit Arslan and Sarikaya’s description (2022) [40].

Therefore, extracting specific data on the RHC here was problematic. However, the direct superior relation of the RHC with the left RA is of the utmost importance during different surgical and endovascular approaches to the left renal pedicle. If it is overlooked, one of the two vessels, or both, could be exposed to iatrogenic trauma and significant bleeding.

Renal transplantation is the best therapeutic approach in end-stage renal disease [41]. With a growing shortage of cadaveric kidneys, live donor kidneys became a critical option to circumvent this issue [41]. Laparoscopic living donor nephrectomies are mainly used [41]. It is essential to avoid injury to the posterior tributaries of the LRV during left living donor nephrectomy [41]. Unfortunately, the posterior tributaries of RVs are not listed in the *Terminologia Anatomica* [42]. In 7/120 (5.83%) cases, LRV anomalies were found during laparoscopic donor nephrectomies: RLRV (one case), CLRV (five cases) and one case of drainage of the LRV in the HAzV [43]. The last case is a scarce variant we did not encounter, in which the RHC is the exclusive pathway for left renal venous blood drainage, and the caval end of the LRV is absent. Laparoscopic donor nephrectomy requires precise preoperative vascular mapping evaluating the RA, the status of the donor kidney and collecting system, and the anatomic definition of the renal venous system [44].

The lumbar veins, if injured, might retract into the retroperitoneal fat in the deepest surgical field with resultant bleeding, causing great difficulty for surgeons in achieving hemostasis [41]. In retroperitoneal laparoscopic nephrectomies, injury to lumbar veins might result in significant complications, including hemorrhage, transfusion, open conversion, and gas embolism [41]. The risk of complications increases when common lumbar-hemiazygos trunks are inserted into the LRV, as we found here.

Yi et al. (2012) found in their meta-analysis that the incidences of communicating veins between the LRV and retroperitoneal veins range from 30.0 to 84.2% in cadaver dissections (median of 68.7%) and from 34.0 to 75.8% in clinical reports (median of 57.5%) [45]. Seemingly, the most common variations in LRV anatomy are the CLRV, RLRV, and those of the RP [45]. We found all these variants possibly associated with RHCs. Although the authors distinguished different anatomical connections of the LRV with lumbar veins, ALV, capsular veins, and HAzVs, the incidence of the RHC was not explicitly evaluated. The LRV may also communicate with the superior mesenteric, gastric, adrenal, and diaphragmatic veins [40]. These communications may also result from errors in the regression process of anastomoses [40]. An RP should be carefully documented preoperatively before approaching of the ureteropelvic junction.

Baniel et al. (1995) studied 72 patients and found, in 43% of the cases, a lumbar vein or veins entering the LRV, usually posterior to the entrance of the left gonadal vein [46]. A similar LRV insertion of the RHC was found in the present study. Baniel et al. (1995) did not observe or record such a vein connecting the LRV and the HAzV. Such details are important because the surgical identification of the typical left gonadal vein may be used to identify a posterior tributary of the LRV, either the RHC or a lumbar vein.

Li et al. (2011) studied 61 cases of retroperitoneoscopic left living donor nephrectomy with anatomical variations of the posterior lumbar tributaries of the LRV [41]. They distinguished seven different anatomical types of the posterior tributaries of the LRV and used the term “lumbar azygos vein”, corresponding to the RHC we studied here. Therefore, the morphological possibilities of the posterior lumbar tributaries of the LRV were found as follows: in type 1 (29.5%)—a lumbar vein; in type 2 (16.4%)—an RHC; in type 3 (16.4%)—separate lumbar vein and RHC; in type 4 (16.4%)—common hemiazygo-lumbar (lumbar-hemiazygos) trunk (we found this variant in 2/11 RHC-positive cases); in type 5 (16.4%)—absent lumbar vein and RHC; in type 6 (1.6%)—lumbar-gonadal trunk; and in type 7 (3.3%)—two lumbar veins and an RHC inserted separately into the LRV [41]. One may observe that although, as a general rule, the standard venous return of the left kidney is supplemented by the reno-hemiazygo-lumbar arch (left supracardinal system) [47], the anatomical backup pathway of this RHC occurs in a few cases.

In patients with a nutcracker or LRV phenomenon/syndrome, the LRV appears narrowed, and the aorto-mesenteric distance is decreased, as is the angle of the superior mesenteric artery [48]. In anterior nutcracker syndrome, the preaortic LRV is compressed. In posterior nutcracker syndrome, either an RLRV or the retroaortic arm of a CLRV is compressed. When the LRV is compressed, the left renal blood flow is redirected through different affluents of the LRV, such as the left gonadal vein, which we found dilated in one case with a narrowed LRV. Posterior affluents of the LRV, which include the RHC, are of such use. All the posterior affluents of the LRV indicate its potential capacity to act as a connecting pathway between the inferior and superior venae cavae [12]. However, although the persistence of LRV hypertension probably causes the development of collateral veins, the nutcracker syndrome also exists in patients in whom angiography shows no collateral veins [49]. Collateralization of the circulation (HAzV, left superior lumbar vein) and congestion during nutcracker syndrome may rarely lead to a spinal epidural hematoma [50]. Therefore, the venous return of the left kidney should be investigated in such cases [50].

The term “tronc reno-rachidien” (renal-rachidian trunk) indicates a large tributary connecting the LRV with the HAzV [51,52]. We also found the term “reno-spinal trunk” [53]. These terms correspond to the RHC we studied here. Certain diffuse forms of myelitis may be due to raised intraspinal venous pressure, resulting in veritable “varicose veins of the spinal cord” [53]. This increased pressure may be due to venous blood reflux from the left kidney into the intraspinal plexuses via the reno-spinal trunk [54]. On the other hand, stenosis of the LRV, third lumbar veins, and the RHC (reno-spinal trunk) determines the stasis of spinal cord veins [54]. Therefore, ligation of the RHC may improve the neurological syndrome resulting from reno-spinal venous reflux [53]. Such ligation was used in two cases of paraplegia due to an inundation of the intrarachidian plexus by venous blood from the LRV, as it brought a marked regression in the paraplegia [52].

Among the RHC cases we documented, we found triple left RAs. A recent meta-analysis regarding the anatomical variants of RAs found a pooled prevalence for triple left RAs of 2.17% [8]. The possible direct relation of the left RA with an RHC has not been documented previously. It is, however, critical during surgical procedures to target the aorta and the left renal pedicle. Preoperative knowledge of the presence of vascular anomalies facilitates the safe performance of aortic surgery [34]. A case with an RHC should also be checked for common renal vessel variations.

One of the limitations of this study is that it focused on the RHC and did not investigate the left reno-lumbar trunks in the non-RHC cases. Lumbar veins are variable and deserve future specific study.

## 5. Conclusions

If present, the RHC serves to connect the superior and inferior caval systems. This may be physiologically of use or not, but surgically, it is a major anatomical risk factor for bleeding if its presence is not checked preoperatively. There are two distinctive types of RHCs, with direct or indirect hemiazygos flow. They can be accurately observed using angioCT scans and three-dimensional volume renderings, and should not be ignored during the anatomical training of students, radiologists, and surgeons. The anatomical variants of the left renal pedicle should be investigated preoperatively, on a case-by-case basis.

## Figures and Tables

**Table 1:** Count and prevalence of types of reno-hemiazygos connection by gender. M: male (N = 4/85 cases); F: female (N = 10/65 cases).

	Type 1	Type 2
M	2/4 (50%)	2/4 (50%)
	2/85 (2.35%)	2/85 (2.35%)
F	9/10 (90%)	1/10 (10%)
	9/65 (13.84%)	1/65 (1.53%)

**Table 2:** Anatomical patterns of left renal veins inserting the reno-hemiazygos connection types (no. of cases, prevalence). LRV: typical preaortic left renal vein; LRV/RP: junction of the typical left renal vein with its retropelvic tributary; RLRV: retroaortic left renal vein; and CLRV: circumaortic left renal vein.

	LRV	LRV/RP	RLRV	CLRV
Type 1 (11 cases)	7 (63.64%)	1 (9.09%)	1 (9.09%)	2 (18.18%)
Type 2 (3 cases)	2 (66.67%)	–	–	1 (33.33%)

**Table 3:** Vertebral levels of the inferior ends of the RHCs, by types (no. of cases, prevalence).

	L1/L2 Disc	L2	L2/L3 Disc	L3
Type 1 (11 cases)	1 (9.09%)	5 (45.46%)	3 (27.27%)	2 (18.18%)
Type 2 (3 cases)	–	1 (33.33%)	–	2 (66.67%)

## Data Availability

Data unavailable due to ethical restrictions.

## Supplementary Materials

The following supporting information can be downloaded at https://www.mdpi.com/article/10.3390/diagnostics15040441/s1: Video S1: Type 1 reno-hemiazygos connection and circumaortic left renal vein.

## Abbreviations

The following abbreviations are used in this manuscript:

ALV	ascending lumbar vein
CLRV	circumaortic left renal vein
CT	computed tomography
HAzV	hemiazygos vein
LRV	left renal vein
MR	magnetic resonance
MRI	magnetic resonance imaging
RHC	reno-hemiazygos connection
RLRV	retroaortic left renal vein
RP	retropelvic tributary
RA	renal artery
RV	renal vein
